# Feasibility-guided evolutionary optimization of pump station design and operation in water networks

**DOI:** 10.1038/s41598-025-17630-w

**Published:** 2025-10-02

**Authors:** Thalía Faúndez-Lizama, Jimmy H. Gutiérrez-Bahamondes, Nicolás Gajardo-Sepúlveda, Daniel Mora-Meliá

**Affiliations:** 1 Master’s Program in Operations Management, Faculty of Engineering, Universidad de Talca, 3340000 Curicó, Chile; 2 Department of Computer Science, Universidad de Talca, 3340000 Curicó, Chile; 3 Department of Hydraulic Engineering and Environment, Universitat Politècnica de València, 46022 Valencia, Spain

**Keywords:** Pumping stations, Data-driven evolutionary optimization, Feasibility predictor model, Machine learning, Classification, Computational science, Computer science, Civil engineering

## Abstract

Pumping stations are critical elements of water distribution networks (WDNs), as they ensure the required pressure for supply but represent the highest energy consumption within these systems. In response to increasing water scarcity and the demand for more efficient operations, this study proposes a novel methodology to optimize both the design and operation of pumping stations. The approach combines Feasibility-Guided Evolutionary Algorithms (FGEAs) with a Feasibility Predictor Model (FPM), a machine learning-based classifier designed to identify feasible solutions and filter out infeasible ones before performing hydraulic simulations. This significantly reduces the computational burden. The methodology is validated through a real-scale case study using four FGEAs, each incorporating a different classification algorithm: Extreme Gradient Boosting, Random Forest, K-Nearest Neighbors, and Decision Tree. Results show that the number of objective function evaluations was reduced from 50,000 to fewer than 25,000. Additionally, The FGEAs based on Extreme Gradient Boosting and Random Forest outperformed the original algorithm in terms of objective value. These results confirm the effectiveness of integrating machine learning into evolutionary optimization for solving complex engineering problems and highlight the potential of this methodology to reduce operational costs while improving computational efficiency in WDNs.

## Introduction

In the past twenty years, water scarcity has become a global concern, reaching critical levels in certain regions of the world^1,2^. According to the United Nations, more than 2.4 billion people lived in water-stressed countries, primarily due to population growth, climate change, and inefficient resource management^3,4^. Acute water scarcity has been documented in several regions, including the Middle East and North Africa (MENA), parts of South Asia, and central Chile, where recurring droughts and overexploitation of aquifers have exacerbated supply-demand imbalances^5,6^. This scenario highlights the urgency of addressing water sustainability challenges and optimizing systems that ensure its distribution. In this context, computationally efficient optimization methods are essential to accelerate the identification of cost-effective design and operation strategies, especially in regions facing acute resource constraints. Moreover, when optimization techniques are applied to real-scale networks, model complexity increases significantly, and traditional evolutionary algorithms often struggle to deliver high-quality solutions within acceptable computational times. These challenges have prompted the development of hybrid and data-driven approaches that enhance performance and scalability^6^.

Water Distribution Networks (WDN) are crucial infrastructures in society, as they ensure equitable access to drinking water. These networks comprise highly complex systems, where their design, operation, and rehabilitation are critical areas of research^7^. One of the main challenges in WDNs is the high energy consumption^8^, specially in direct injection networks^9^. Previous works show that pumping stations (PSs) are responsible for the highest energy consumption in the system^10,11^, as they supply the energy needed to drive water through the pipes to the consumption points. Therefore, optimizing PSs is essential for enhancing network efficiency^12,13^, as studies have shown that these stations significantly reduce energy consumption and costs^14^.

The optimization of PSs involves two approaches^9^. The first is the design approach, which focuses on aspects such as the location of PSs^13^, the selection of pump models based on capacity^15^, and optimizing the combination of the number of pumps and their capacity^16^, among others. The second approach is operational, which includes pump scheduling^7,17^, cost reduction in supplying variable flows^18^, and the scheduling of fixed-speed and variable-speed pumps^19^. Some authors have even attempted to optimize both approaches simultaneously; however, this can greatly increase the complexity of the models^20–22^.

Efficient methods are required to solve these optimization models. Evolutionary algorithms (EAs) have proven to be effective tools for tackling highly non-linear problems, with genetic algorithms (GAs) standing out^8,23^. These metaheuristics have been applied in WDNs to address issues suchs as minimizing water loss^24^, maximizing hydraulic system performance while reducing operational and maintenance costs^25^, optimizing the design of a WDN using a hydraulic simulator^26^, and conducting multiobjective optimization of operational costs, water supply equity, and pressure level uniformity across the network^27^. Along these lines, a penalty-free evolutionary approach was proposed^28^, which simultaneously integrates tank siting and sizing, pump scheduling, and water quality analysis through pressure-driven extended-period simulations, yielding feasible and hydraulically efficient solutions with competitive costs. For EAs to function properly, optimizing their hyperparameters is crucial, although it can be challenging^29^.

A clear example of this is a study that minimizes both investment and operational costs of pumping stations^21^ through a nonlinear programming model solved using a Pseudo-Genetic Algorithm^30^.Verifying the fulfillment of model constraints is the most complex task, as it requires at least one hydraulic simulation for each analysis period. If leaks are considered, the number increases^31^. In real-world networks with thousands of components, computational effort becomes critical due to the vast solution space, where infeasibility predominates. Consequently, as the scale and complexity of the problems increase, the effectiveness and applicability of optimization methods become limited^32^.

To address computational effort challenges, some researchers have focused on reducing the search space, highlighting feasibility analysis in optimization, which accelerates both convergence and solution evaluation. Solution clustering has also been applied in various contexts, such as pipe sizing based on topological and hydraulic metrics^33^, feasibility evaluation of pump scheduling in hydraulic simulators^34^, and identifying feasible regions when applying mathematical models for energy reduction^35^. In the context of PSs in WDNs, methodologies have been developed to assess the feasibility of solutions, applying hierarchical analysis based on technical and economic criteria for the proper selection of pumps^36,37^. A bi-phase algorithm has also been employed for pump scheduling, creating a feasible solution and then refining it^38^, along with the development of infeasibility maps^39^, to exclude infeasible areas during the search process.

Another approach to optimization involves the use of surrogate or machine learning (ML) models^40^, which can include classification or regression models^41^. Some ML applications in complex infrastructures include the detection and localization of structural damage using convolutional neural networks^42,43^ and the development of models to classify and locate defects in sewer pipelines^44^. In WDNs, these models have been applied in various ways, such as failure prediction^45^, detecting pipe failures using Artificial Neural Networks (ANN)^46^, and the detection of events related to water quality parameters using SVM^47^. Other examples include enhancing the optimization process of genetic algorithms by using a trained model^48^, and replacing hydraulic simulations with ANN-based approximations^49^. Despite the success of these applications, there are three main limitations in the application of surrogate models: high dimensionality, which makes their application in real-world scenarios; their deterministic black-box nature, which limits transparency and applicability; and their rigid architecture, which restricts their generalization across different case studies^50^.

Currently, the data-driven evolutionary optimization methodology is emerging, which uses surrogate algorithms trained with historical data to predict the performance of specific parameter configurations^51^, supporting the optimization process of evolutionary algorithms. Data-driven evolutionary algorithms (DDEA) are effective in solving expensive real-scale optimization problems, achieving satisfactory solutions with a limited number of evaluations of the problem’s objective function^52^. DDEA can operate online, where they evaluate new sample points and continuously improve the accuracy of the surrogate model, or offline, where such feedback does not exist, making the quality and representativeness of the training database critical^53^.

Several studies have explored this methodology, although their focus is not on specific applications within civil engineering. This limited adoption is partly due to barriers such as the lack of integration between machine learning frameworks and widely used hydraulic simulation software, which complicates the deployment of hybrid workflows. Moreover, data-driven models typically require large volumes of labeled data, which are not readily available for most real water infrastructure systems. Finally, the implementation of these approaches demands a combination of domain-specific knowledge and advanced data science skills, which are not yet widespread in the civil engineering community. Instead, of focusing on civil engineering applications these studies aim to enhance training procedures for predictive models. For instance, some approaches support an evolutionary algorithm with a set of surrogate models integrated through ensemble learning, thereby optimizing local accuracy^51^. Similarly, a tri-training approach using radial basis function networks (RBFN) as surrogates has been proposed, where the models are dynamically updated through pseudo-labels automatically generated during optimization^53^.

The application of data-driven evolutionary optimization (DDEO) in WDNs is extremely limited, whereas most reported studies have focused on urban drainage systems. For instance, surrogate model–assisted evolutionary algorithms have been developed to optimize the design of stormwater networks^54^ and the operation of flow control and storage infrastructures during extreme rainfall events^55^. This greater adoption in the drainage domain can be explained by operational differences (free-surface flow versus continuous pressurized flow in WDNs), greater availability of historical or inspection data in drainage systems, and the strict regulatory and continuous service requirements characteristic of WDNs, which hinder the calibration, transferability, and application of surrogate models in such networks^56,57^.

The main contribution of this study is the development of a novel DDEA, which evaluates only those solutions classified as feasible by the already trained ML model inside of each evolutionary process, significantly reducing the computational effort associated with hydraulic simulations. A real case study was developed achieving a reduction in the number of objective function evaluations from 50,000 to less than 25,000, while also obtaining higher-quality solutions compared to traditional methods. These results underscore the computational efficiency of the proposed methodology and its potential for broader applications in civil engineering and water resource management.

The remainder of this article is structured as follows: Section 2 describes the materials and methods, including the proposed approach, the integrated machine learning classifiers, and the case study. Section 3 presents and analyzes the results, comparing the performance of the different methods and discussing their computational implications, highlighting the potential of the proposed methodology to reduce computational effort. Finally, Section 4 summarizes the key contributions and outlines potential directions for future research.

## Methods

This study extends the previously proposed methodology^21^ to a real-scale application using the DDEO (Data-Driven Evolutionary Optimization) approach.The section presents the mathematical model governing the optimization process, the decision variables and objective function, the constraints applied to the model, and the process for evaluating the feasibility of solutions. Additionally, it details the integration of a Feasibility Predictor Model (FPM) into the evolutionary optimization process, the application of the methodology to a real-world case study, and the computational tools used for its implementation.

### Mathematical model

For a better understanding of the problem, this section presents a previously proposed mathematical model aimed at optimizing the operational and investment costs of pumping stations (PS)^21^. The model is based on the setpoint curve (SC)^58^, which defines the minimum head required by each pumping station to meet the demand. The content presented below is a summarized version of the mathematical model, with a detailed description available in the original study^21^.

The model considers several hydraulic parameters essential for the optimization process. These include the total time steps $N_t$ and the total number of PSs in the network $N_{\text {ps}}$, as well as the number of available pump models $N_b$ in the dataset. Each pumping station (PS) contains a defined number of pumps $NB_i$, which are characterized by head coefficients $( H_{\text {0,i}}, A_i )$ and efficiency curves $(E_i, F_i)$. The model also incorporates operational constraints, such as the maximum number of pumps per PS $P_{\text {max}}$, the maximum pump head $H_{B_{\text {max}}}$, and the head supplied by each station $H_{\text {max}, i}$. Furthermore, it distinguishes between fixed-speed pumps $m_{\text {i,j}}$ and variable-speed pumps $n_{\text {i,j}}$, with the latter being supported by frequency inverters $n_i$. Additional parameters include the specific weight of water $\gamma$ and the division of analysis periods into discrete time intervals $\Delta _{\text {t,j}}$.

The model incorporates several economic parameters essential for assessing project viability. It considers the number of lifecycle periods $(N_p)$ and the amortization factor $( F_a )$, which accounts for the interest rate $r$ over time. Additionally, the model includes cost components such as energy costs $(p_{i,j})$, pump acquisition costs $(Cpump_i )$, accessories$(Cfacility_i )$ and control tools $(Ccontrol_i )$. This comprehensive approach ensures an accurate economic evaluation of the pumping station system while balancing efficiency and sustainability.

This integrated approach ensures accurate modeling of PS performance, considering both hydraulic efficiency and economic sustainability.

#### Decision variables

The optimization model determines the following decision variables:

$X_{{\text {i,j}}}$: Percentage of the flow supplied by $P_{S_{\text {i}}}$ at time step j.

$m_i$: Number of fixed-speed pumps in $P_{S_{\text {i}}}$.

$b_i$: Pump model ID to be installed in $P_{S_{\text {i}}}$, within the range [1, MB]

#### Objective function

The optimization seeks to minimize total project costs, including both capital $CAPEX$ and operational $OPEX$ costs. Equation (1) defines the total project cost by incorporating the amortization factor $(F_a)$, which accounts for the interest rate $r$ over $N_p$ periods.

1 $$\begin{aligned} F= & F_a \cdot \text {CAPEX} + \text {OPEX} \end{aligned}$$

2 $$\begin{aligned} F_a= & \frac{r \cdot (1 + r)^{N_p}}{(1 + r)^{N_p} - 1} \end{aligned}$$

The capital costs (CAPEX) and operational costs (OPEX) are calculated using equations (3) and (4), respectively. Equation (5) presents the calculation of the parameter $\alpha$, which enables pump control by adjusting their heads to the setpoint curve. This adjustment, in turn, is essential for determining the operational costs.

3 $$\begin{aligned} \text {CAPEX} = \sum _{i=1}^{N_{\text {ps}}} \left( N_{B_i} \cdot C_{\text {pump}, i} + n_i \cdot C_{\text {inv}, i} + C_{\text {facility}, i} + C_{\text {control}, i} \right) \end{aligned}$$

4 $$\begin{aligned} \text {OPEX} = \sum _{j=1}^{N_t}\left\{ \sum _{i=1}^{N_{\text {ps}}} \left[ \sum _{k=1}^{m_{ij}} \frac{\omega \cdot (H_{0i} - A_i \cdot Q_{ijk}^2 )}{(E_i - F_i \cdot Q_{ijk} )} + \sum _{k=1}^{n_{ij}} \frac{\omega \cdot (H_{0i} \cdot \alpha _{ijk} - A_i \cdot Q_{ijk}^2 )}{E_i / \alpha _{ijk} - F_i / \alpha _{ijk}^2 \cdot Q_{ijk} } \right] \cdot P_{ij} \right\} \Delta _{t_j} \end{aligned}$$

5 $$\begin{aligned} \alpha = \sqrt{\frac{H_{S_{ij}} + A_i \left( \frac{Q_{ijk}}{N_{B_i}} \right) ^2}{H_{0i} }} \end{aligned}$$

The parameter $\alpha$ in Equation (5) represents the speed adjustment factor for variable-speed pumps, reflecting how much the pump speed must deviate from its nominal operating condition to fulfill specific hydraulic requirements defined by the SC. Specifically, $\alpha =1$ indicates operation at nominal speed, $\alpha <1$ indicates reduced speed operation (lower head and flow, resulting in energy savings), and $\alpha >1$ indicates increased speed operation (higher head and flow at the expense of increased energy consumption). Thus, $\alpha$ directly influences energy efficiency by dynamically matching pump operations to varying hydraulic demands.

#### Constraints

The constraints in this model define the permissible values for the decision variables, ensuring that the system operates within realistic and practical boundaries. Equations (6) and (7) establish that the total demand flow must be fully supplied by the PSs; however, not all stations are required to operate during every time period. Equation (8) ensures that the selected pump has a head capacity exceeding the maximum required head to guarantee proper supply. Finally, Equation (9) imposes an upper limit on the number of pumps that can be installed at each PS.

6 $$\begin{aligned} x_{ij} \ge 0 \quad \forall _{ij} \end{aligned}$$

7 $$\begin{aligned} \sum _{i=1}^{N_{\text {ps}}} x_{ij} = 1 \quad \forall _{j} \end{aligned}$$

8 $$\begin{aligned} H_{0i} \ge H_{\text {max}, i} \quad \forall \text { PS}_i \end{aligned}$$

9 $$\begin{aligned} P_{\text {max}} \ge N_{B_i} \quad \forall \text { PS}_i \end{aligned}$$

### Objective function evaluation (OFE)

To effectively address the optimization problem, it is essential to evaluate solutions on a large scale using an optimization algorithm. However, the evaluation process is the most computationally intensive stage, as each OFE requires at least one hydraulic simulation for every analysis period. In real-world scenarios, a single hydraulic simulation may involve assessing water supply to thousands of demand points, significantly increasing computational effort.

**Fig. 1:**
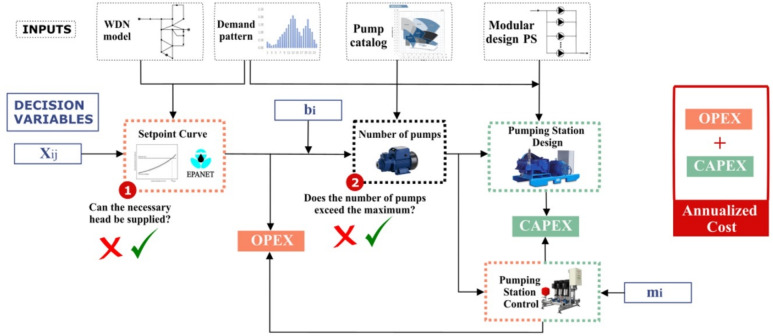
Interactions in an OFE.

Moreover, due to the complexity of the problem, an OFE not only involves hydraulic simulations but also multiple interdependent calculations that interact to produce the final result. These calculations determine key cost components, specifically OPEX and CAPEX, which are critical for assessing the feasibility and efficiency of a solution. The interactions within the evaluation process are illustrated in Fig. 1.

Once all hydraulic simulations and cost calculations are completed, the feasibility status and total cost of each solution can be determined. However, many candidate solutions turn out to be infeasible and are subsequently discarded from the study. This inefficiency results in a substantial waste of computational resources, making the process both time-consuming and costly. To address this issue, this study proposes the implementations of a Feasibility Predictor Model (FPM), which optimizes the evaluation process by filtering out infeasible solutions before conducting extensive simulations, thereby reducing computational burden and enhancing efficiency.

### Feasibility Predictor Model (FPM)

**Fig. 2:**
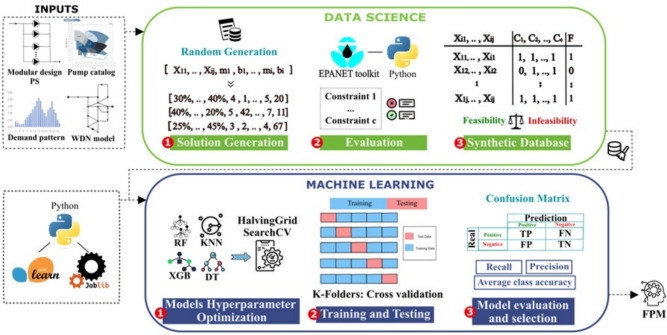
FPM Formation.

To enhance the efficiency of the optimization process, this study proposes the development of a Feasibility Predictor Model (FPM), which classifies solutions based on their feasibility before performing detailed evaluations. By acting as a pre-filter, the FPM identifies and discards infeasible solutions early in the process, significantly reducing the number of evaluations required and thereby decreasing computational effort. The proposed methodology is illustrated in Fig. 2.

To implement the FPM, essential input data is required to accurately contextualize and solve the problem. This includes a complete WDN model with details such as pipe lengths, diameters, and node elevations, a comprehensive pump catalog with model specifications, demand patterns for each analysis period (typically 24 hours), and a modular design for pumping stations.

#### Step 1: Synthetic Data Generation

The first step involves generating synthetic data to create a labeled database composed of solutions classified by their feasibility. Each solution comprises two components: an operational component, which describes the demand flow distribution across PSs for each time period, and a design component, specifying the number of variable-speed pumps and the selected pump model for each PS.

**Fig. 3:**
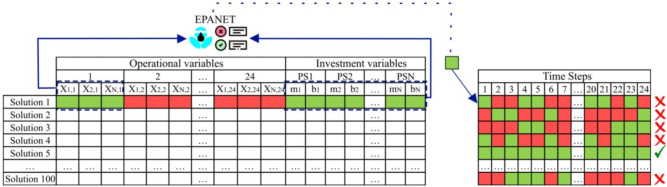
Period-by-period analysis.

To build the synthetic database, random solutions are generated by varying pump configurations and flow distribution. Each configuration is then evaluated using the hydraulic simulator EPANET, which assesses their feasibility across multiple analysis periods. The feasibility evaluation consists of three key criteria: (1) verifying compliance with pressure requirements based on the setpoint curve, (2) ensuring the selected pump model has sufficient head capacity to meet supply demands, and (3) confirming that the total number of pumps does not exceed predefined constraints. If all conditions are met for a given period, it is considered feasible. A solution is deemed fully feasible only if all its periods satisfy these constraints; otherwise, it is labeled as infeasible. Figure 3 illustrates this period-by-period analysis.

Specifically, a candidate solution is considered to meet the pressure compliance requirement if the pressure head at every demand node *i* in the network and at each time step $t \in \{1, \dots , 24\}$ satisfies the following condition:

10 $$\begin{aligned} H_i(t) \ge H_{\text {min}} = 20\, \text {mwc} \end{aligned}$$

where $H_i(t)$ is the pressure head (in meters of water column) at node *i* at time *t*, and $H_{\text {min}}$ is the minimum allowable pressure threshold, set to 20 mwc in this study. This constraint is enforced across all demand nodes and all time steps in the 24-hour simulation horizon.

This structured approach allows differentiation between solutions based on their feasibility levels. Additionally, it refines the training process by preventing the model from being influenced by an excessive number of parameters.

Once the database is structured, it includes the flow distribution percentages for each PS, the demand pattern for each period, and the characteristics of the selected pump model for each station (the maximum head and flow it can provide), along with the corresponding feasibility classification. Standardization of flow distribution values is not required, but class balancing techniques are applied to improve model performance.

#### Step 2: Preprocessing and model training

To effectively address the classification problem, multiple ML models were evaluated to identify those with the highest predictive accuracy and reliability. This step involves fine-tuning hyperparameters to optimize model performance, training the models offline using the processed dataset, and rigorously validating them with various evaluation metrics. A systematic approach is followed to ensure an optimal balance between computational efficiency and prediction quality. The process of training the FPM is detailed in Algorithm 1.

**Algorithm 1:**
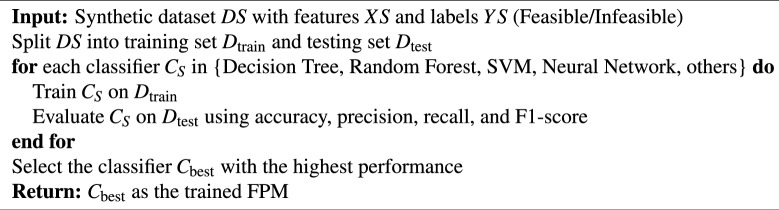
Train Feasibility Predictor Model (FPM)

### Data-driven evolutionary optimization with FPM

The DDEO methodology aims to reduce the computational effort of evolutionary optimization processes by integrating a ML model. The general framework of this methodology combines the fields of data science, machine learning, and evolutionary algorithms. The interaction among these fields involves using problem-related data, whether historical or generated through simulations, to train an ML model capable of learning patterns from these parameter configurations. Once trained, this model is integrated into the evolutionary algorithm, either as a surrogate model that replaces certain calculations in the optimization process or as a support tool for specific tasks.

#### Step 3: Evolutionary algorithm guided by FPM

Once the FPM has been trained, it is integrated into the evolutionary optimization process to enhance solution evaluation. The trained model is used to classify candidate solutions as feasible or infeasible before performing computationally expensive hydraulic simulations. This predictive filtering allows the evolutionary algorithm to focus on promising solutions while discarding unfeasible ones early in the process.

During the optimization process, each generated solution is first analyzed by the FPM. If the model classifies a solution as infeasible, it is assigned a penalty that directs the search towards more viable alternatives. The evaluation process begins by querying the model whenever a solution requires assessment. Conversely, if the FPM determines that a solution is feasible, it proceeds to full hydraulic simulations for further refinement. This Feasibility-Guided Evolutionary Algorithm (FGEA) significantly reduces computational costs and enhances optimization efficiency. The proposed methodology is illustrated in Algorithm 2.

**Algorithm 2:**
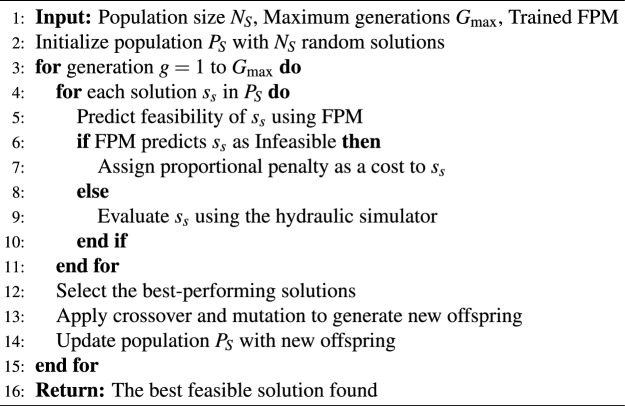
FGEA

By implementing this methodology, the FPM streamlines the optimization process, reducing computational load while ensuring that only promising solutions are considered for further evaluation. To enhance the efficiency of the optimization process, this study proposes the development of a FPM, which classifies solutions based on their feasibility before performing detailed evaluations. By acting as a pre-filter, the model identifies and discards infeasible solutions early in the process, significantly reducing the number of evaluations required and thereby decreasing computational effort.

### Case study

The Curicó network, located in central Chile, was selected as a case study due to the availability of a calibrated hydraulic model and access to operational data through collaboration with the local water utility. This real-world system reflects the complexity of medium-sized urban networks, featuring elevation differences, pressure constraints, and multiple pumping stations. These attributes present realistic operational challenges that make it particularly suitable for evaluating the proposed optimization methodology under practical conditions.

Standard benchmark networks such as Anytown or Hanoi are commonly used for methodological validation; however, they are relatively small and do not capture the full range of hydraulic and topological complexity found in operational water distribution systems. In contrast, the Curicó network enables a more rigorous and representative assessment of computational performance and feasibility prediction in a real-world context.

**Fig. 4:**
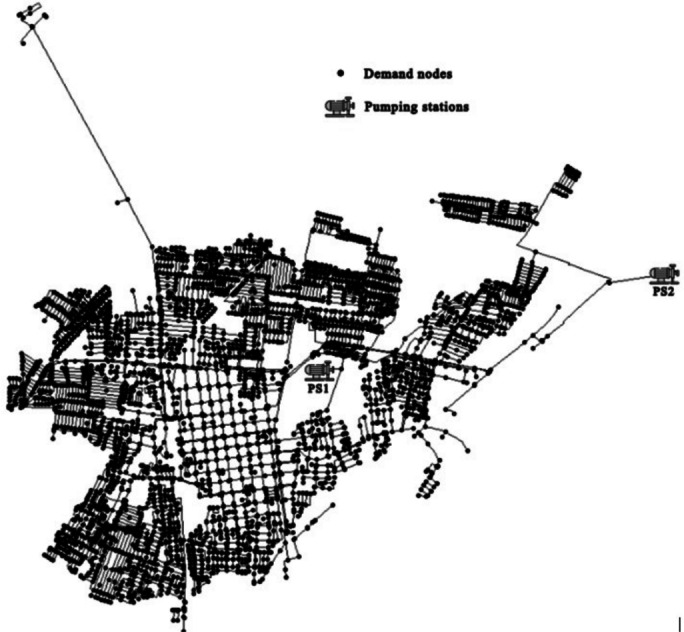
Curicó network.

Figure 4 shows the topology of this network, which comprises 7,362 demand nodes, 8,358 pipes and two PSs. The analysis is conducted over 24-hour period, with each hour modeled as a discrete time step in the optimization process. The baseline demand for the network is 170 liters per second (L/s).

The minimum pressure requirement at the nodes is set to 20 meters of water column (mwc), a crucial constraint to guarantee proper water distribution throughout the system. To address the optimization problem, a catalog containing 67 different pump models is considered, allowing for a broad selection of possible configurations. The optimization aims to determine the optimal number and type of pumps to install at each PS while ensuring compliance with hydraulic and economic constraints. The Curicó network serves as a practical validation scenario for assessing the computational efficiency and effectiveness of the FPM in guiding the evolutionary optimization process.

### Computational implementation

The entire optimization framework is implemented in Python, leveraging multiple libraries to ensure robust data processing, machine learning model training, and evolutionary algorithm execution. The database used to train the ML models is created by evaluating 500 candidate solutions, which are generated and assessed based on their feasibility within the Curicó network. This sample size was selected to balance predictive model quality and computational feasibility. Preliminary tests with larger datasets yielded marginal gains that did not justify the added simulation cost, while smaller datasets led to reduced generalization and poorer optimization performance. The selected configuration enabled reliable prediction of solution feasibility and consistent convergence to high-quality results in more than 80% of the experiments.

For data preprocessing, the Panda´s library is utilized to structure and manage large datasets, while Scikit-learn is used for class balancing via the undersampling method, ensuring a well-distributed dataset for model training. Randomized numbers for solution generation are handled using the Random library. Various machine learning models were trained — including Extreme Gradient Boosting, Random Forest, K-Nearest Neighbors, Decision Tree, Artificial Neural Networks, Logistic Regression, Naive Bayes, and Support Vector Classifier — using the Scikit-learn library, with hyperparameter tuning performed through the HalvingGridSearch method. To ensure robustness and a proper adjustment to identify feasibility, the HalvingGridSearch procedure was executed with different hyperparameter grids for each classification model. Model validation was carried out using K-Folds Cross-Validation with K = 10, and performance was evaluated based on accuracy, recall, and precision metrics. Subsequently, the best-performing models — Extreme Gradient Boosting, Random Forest, K-Nearest Neighbors, and Decision Tree — were selected, considering as the main criterion those that achieved a good accuracy score in testing. Finally, the selected models were exported using the Joblib library for efficient integration into the evolutionary optimization algorithm.

The genetic algorithm (GA) is implemented using the JMetalPy library, which provides a flexible structure for developing evolutionary algorithms. The trained FPM is integrated within this algorithm, acting as a filtering mechanism that prevents the evaluation of infeasible solutions during the optimization process. Each solution is evaluated using a full hydraulic simulation implemented in EPANET 2.2, integrated through a Python interface. The simulation comprises 24 demand periods, during which pressure and flow conditions are verified to ensure feasibility across the network. In the presented case study, the average computational time for evaluating a single solution is approximately 7.53 seconds on a personal computer with an Intel Core i7 processor and 16 GB of RAM. Although this time is relatively low for a single evaluation, the optimization process typically involves thousands or even hundreds of thousands of evaluations. This leads to accumulated computation times that may span weeks or even months, rendering the approach impractical for large or urgent design tasks. This computational cost motivated the introduction of the Feasibility Predictor Model (FPM) to reduce the number of full hydraulic simulations required.

To benchmark performance, 50 independent optimization experiments are conducted using the traditional method to obtain an optimal solution, followed by 50 experiments for each FGEA where each variant differs based on the applied ML-based feasibility filter. In total, 250 experiments are performed to validate the efficiency and robustness of the proposed approach. The final study analyzes the best solutions obtained, as well as the ability to identify good solutions. Good solutions are defined as those whose objective function values slightly exceed the best known value, in accordance with commonly accepted criteria in similar optimization studies^59^. The implementation is carried out in Python, using the Pandas, Scikit-learn, JMetalPy, and EPANET libraries. A total of 250 experiments are conducted, comparing the traditional approach with the proposed methodology enhanced by the FPM.

## Results and discussion

This section presents the results obtained from applying the proposed methodology to the Curicó case study. Four variants of the FGEA approach were trained and validated, each integrating a different FPM. To ensure greater statistical validity of the results, 50 independent experiments were conducted for each FGEA, which were compared against 50 experiments performed using a traditional EA. The primary objective of this comparison is to evaluate the ability of the FGEAs to reduce the computational cost of the optimization process without compromising solution quality.

The stopping criterion of 50,000 solutions generated per experiment was established. The results indicate that the method achieved an optimal cost of €45,947.44. To evaluate the quality of the solutions obtained, a 1% loss threshold was defined relative to the best value found, to classify the so-called “good solutions.” In this context, any solution with an objective function value below €46,406.91 is considered a good solution.

### Cost and optimization performance

**Fig. 5:**
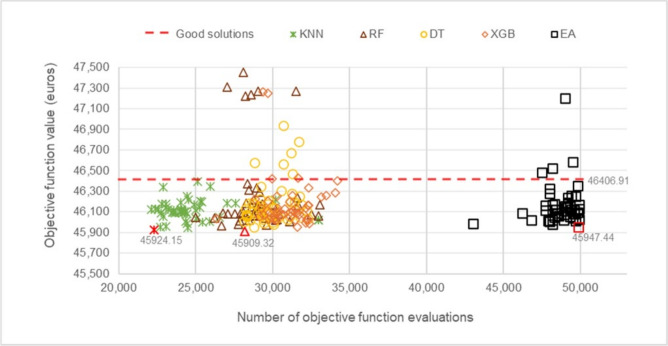
Cost and OFE relationship of the experiments.

Figure 5 presents the cost distribution of the experiments conducted, illustrating the relationship between the objective value obtained and the number of OFEs required to achieve it in each experiment. This representation allows for mapping the results and analyzing the performance of the different proposed methods, facilitating their comparison with the traditional EA.

Regarding the behavior of the OFEs performed by the different methods to obtain the best solution in each experiment (considering a maximum of 50,000 OFEs), it is observed that the EA evaluates between 45,000 and 50,000 solutions, although in some cases it fails to converge on good solutions. In the case of FGEA-RF and FGEA-DT, greater dispersion in the results is observed, as several experiments do not reach good solutions, with OFEs ranging approximately between 25,000 and 35,000 evaluations. This behavior is also observed in FGEA-XGB, which shows a similar pattern in terms of the number of evaluations. Finally, FGEA-KNN evaluates approximately 20,000 to 30,000 solutions in most experiments, achieving the highest savings performance.

To further analyze the performance of each FGEA, Table 1 summarizes the most relevant results obtained with each machine learning model, including the number of “good solutions” generated, the average costs achieved, and the best solution found by each approach. Notably, FGEA-RF obtained the best individual solution, with a cost of €45,909.32 after 28,248 objective function evaluations (OFEs). Meanwhile, FGEA-KNN achieved a similar cost of €45,924.15, requiring only 22,303 OFEs. These results demonstrate that some FGEAs not only significantly reduce computational effort but can also improve solution quality compared to traditional approaches.

**Table 1:** Summary of results for each model.

	Good	Average objective	Best solution		
Method	solutions	value (€)	Objective value (€)	OFEs	**Saving**
EA	84%	46,591.16	45,947.44	50,000	-
FGEA-DT	80%	46,620.79	45,954.03	28,833	42%
FGEA-KNN	94%	46,191.51	45,924.15	22,303	55%
FGEA-RF	82%	46,531.13	45,909.32	28,248	44%
FGEA-XGB	90%	46,283.53	45,948.89	31,669	37%

The findings indicate that all FGEAs were able to generate good solutions in at least 80% of the experiments conducted. Notably, FGEA-KNN and FGEA-RF outperformed the traditional EA, with FGEA-KNN achieving better solutions on two separate occasions. These findings validate the effectiveness of ML-based feasibility filters in guiding the optimization process toward high-quality solutions while significantly reducing computational costs.

### Evaluation of hydraulic performance

This section presents a comparative hydraulic analysis between the best solution from the traditional EA (€45,947.44 with 50,000 OFEs) and the most efficient cost-OFEs solution, obtained through FGEA-RF (€45,909.32 with 28,248 OFEs).

**Fig. 6:**
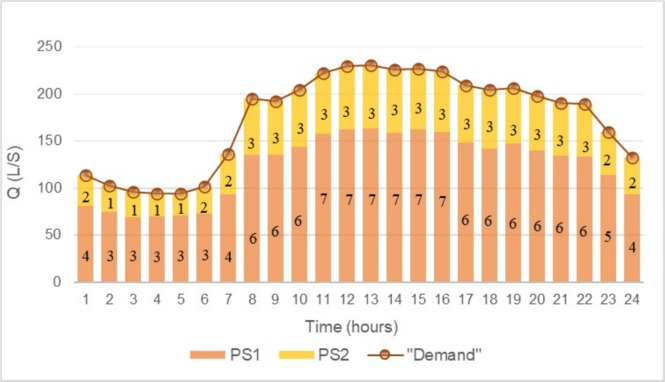
EA optimal solution.

**Fig. 7:**
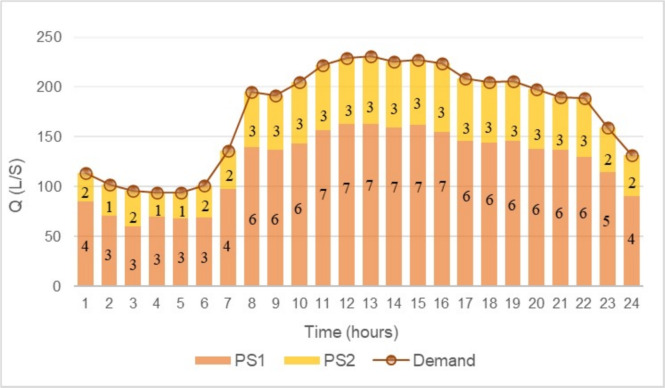
FGEA-RF optimal solution.

Figures 6 and 7 illustrate the pumping scheme behavior over a 24-hour period for both solutions. In these graphs, the bars represent the number of active pumps in each period, while the dotted lines indicate the total flow required by the network.

The results show that both solutions exhibit a highly similar pump scheduling and flow distribution among the PSs. Moreover, for PS1, both solutions display identical behavior throughout the entire analysis period. The main difference lies in the pumping scheme of PS2, where the solution obtained using FGEA-RF operates with one less pump in period 3, leading to a reduction in operational costs. Additionally, the differences in operational costs (OPEX) and investment costs (CAPEX) are analyzed. Table 2 provides a detailed comparison of the total annual costs for both solutions. In terms of CAPEX, no differences are observed between the solutions, as both involve the same infrastructure investment: 7 pumps of the “GNI 50-13/5.5” model in PS1 and 3 pumps of the “GNI 50-13/7.5” model in PS2.

The main difference lies in the operational costs (OPEX), specifically in the variations observed in PS2, which directly impact the total operating cost. As a result, the solution obtained through FGEA-RF emerges as the most cost-effective alternative, optimizing resource utilization without compromising the hydraulic feasibility of the system.

**Table 2:** Cost comparison between the best solutions EA and FGEA-RF.

	OPEX		CAPEX		$\varvec{F_a} \cdot$ CAPEX + OPEX	
	EA	FGEA-RF	EA	FGEA-RF	EA	FGEA-RF
PS1	€ 25,170.2	€ 25,156.7	€ 6,918.2	€ 6,918.2	€ 32,088.4	€ 32,074.8
PS2	€ 10,922.8	€ 10,898.2	€ 2,936.3	€ 2,936.3	€ 13,859.1	€ 13,834.5
Total					**€ 45,947.5**	**€ 45,909.3**

Both solutions meet the hydraulic constraints defined by the problem and the mathematical model; however, the solution obtained through FGEA-RF demonstrates greater efficiency and cost reduction, while also aligning more precisely with the network requirements. This optimization is crucial, as an inefficient exploration of the search space can lead to unnecessary operational and infrastructure costs.

### Analysis of computational effort to obtain good solutions

**Fig. 8:**
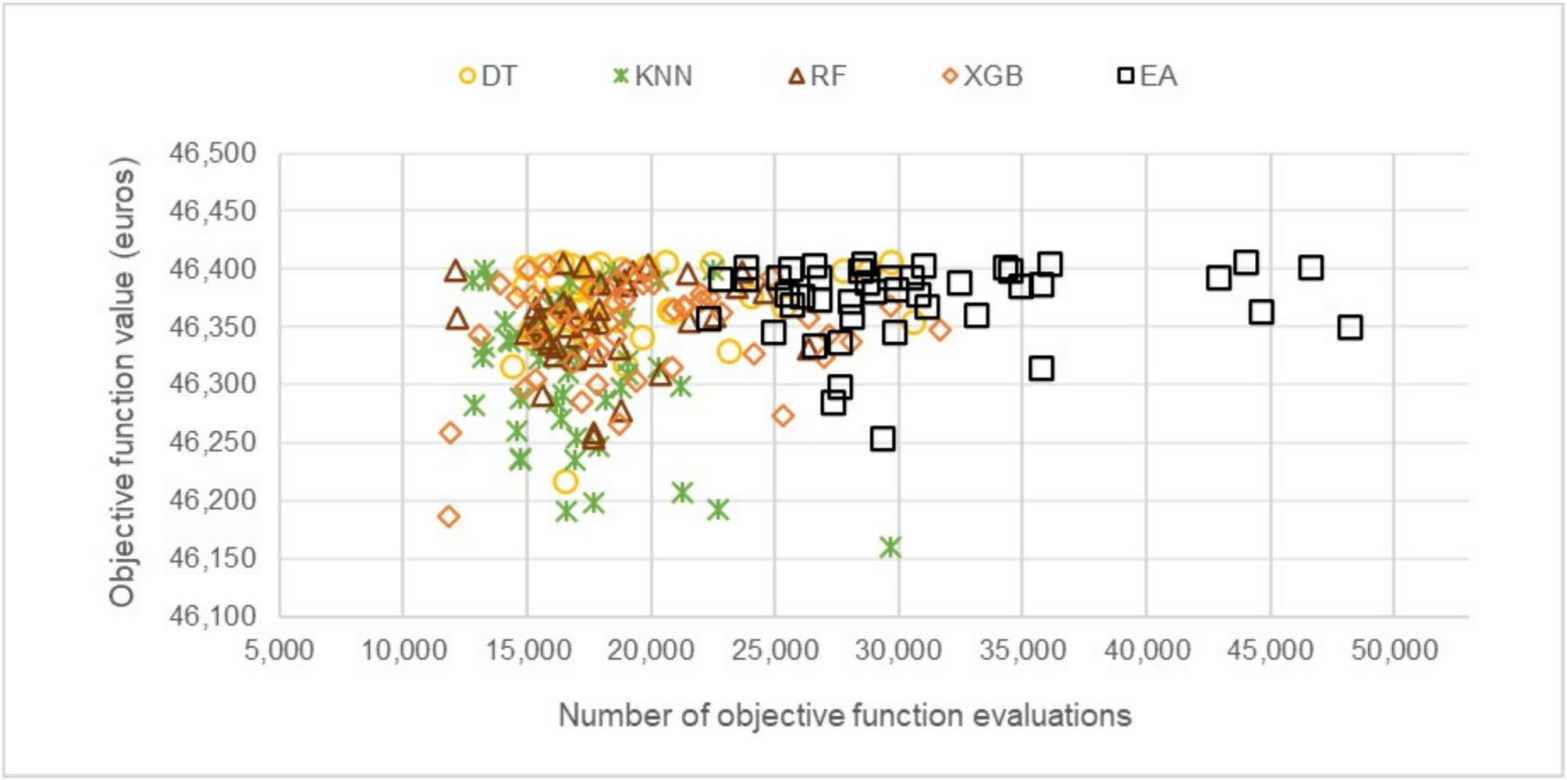
Cost and OFE relationship of the first good solution in each experiment.

One of the key objectives of the proposed methodology is to reduce the number of OFEs required for optimization. Figure 8 shows the number of OFEs needed to obtain the first good solution in each experiment, allowing for a comparison of the computational savings achieved by the different methods.

The distribution shown in Fig. 8 demonstrates that all FGEAs are capable of reducing the number of OFEs required to obtain good solutions compared to the original method. While the EA displays a distribution mainly concentrated between 20,000 and 35,000 OFEs, the FGEAs concentrate their evaluations between 10,000 and 25,000 OFEs, reflecting a significant computational saving. For a more detailed analysis, Table 3 presents a statistical summary of the OFEs required to obtain good solutions.

**Table 3:** Statistical summary of the OFEs required to obtain good solutions.

	EA	FGEA-KNN	FGEA-RF	FGEA-DT	FGEA-XGB
Average	30,660	17,202	18,035	19,018	19,649
Minimum	22,300	12,771	12,088	14,406	11,837
Maximum	48,200	29,654	26,346	30,599	31,684
Median	29,000	16,518	17,708	17,437	18,847
Standard deviation	6,282	3,481	3,062	4,163	4,620

According to the results presented in the previous table, all FGEAs reduce the average number of OFEs required to obtain a good solution by at least 35%. In particular, FGEA-KNN achieves the highest reduction with 44%, followed by FGEA-RF with 41%, FGEA-DT with 38%, and FGEA-XGB with 36%.

From another perspective, when comparing the FGEAs with the EA based on the standard deviation, it is observed that the most optimistic scenario for the EA (one standard deviation below the mean) corresponds to obtaining a good solution with 24,378 OFEs. In contrast, even the most pessimistic scenarios for the FGEAs —that is, one standard deviation above the mean— show better performance: FGEA-KNN requires up to 20,683 OFEs, FGEA-RF 21,097, FGEA-DT 23,181, and FGEA-XGB 24,269. This demonstrates that integrating ML classifiers into evolutionary optimization can lead to substantial computational efficiency improvements, enabling the generation of high-quality solutions at a significantly lower computational cost.

## Conclusions

Water distribution networks (WDNs) are the subject of constant research due to the high complexity associated with their design and operation. While current metaheuristic tools have demonstrated good performance, their efficiency decreases significantly as the dimensionality of the problem increases, leading to high costs and considerable computational effort. In this context, this study proposes a new FGEA that involves integrating a Feasibility Predictor Model (FPM) into an EA. This model classifies the solutions generated by the evolutionary algorithm and allows the evaluation of only those labeled as feasible, thereby reducing the number of hydraulic simulations required in the heuristic optimization process. To construct the FPM, machine learning classification models were employed.

The proposed methodology was implemented and validated in a real-scale case study. The results demonstrated the effectiveness of the approach, as all the classification models used to formulate the FPM achieved significant savings in objective function evaluations (OFE). Specifically, the number of OFEs was reduced from 50,000 to less than 25,000, without compromising the quality of the solutions obtained compared to the original method. Furthermore, in some cases, the optimal cost defined by the original method was improved while reducing the number of evaluations, offering a dual benefit: computational savings and result optimization.

This methodology demonstrates its contribution to the optimization of complex infrastructures, such as WDNs, by offering an innovative approach with high potential for application to other critical infrastructures and possible expansion into new studies. However, since the current model is based on a specific dataset and optimization framework, its adaptability to different hydraulic conditions or larger-scale problems may require additional methodological adjustments. A key limitation of the proposed approach is that the FPM must be trained specifically for each water distribution network. Due to the strong dependency of hydraulic feasibility on network topology, pump configurations, and local demand patterns, the knowledge learned from one case cannot be transferred to another. Consequently, each new network requires a dedicated synthetic dataset and a newly trained model, which may significantly increase the computational cost when analyzing multiple scenarios. Moreover, as the number of pumping stations grows, the dimensionality of the solution space increases, demanding a larger dataset and further complicating model training. These aspects limit the direct scalability and generalization of the methodology across different network designs. Addressing these challenges—by exploring transferable learning models, domain adaptation, or data-efficient training strategies—represents an important direction for future research.

Regarding potential improvements and future developments, this research could evolve into a feasibility prediction model with a linear rather than categorical approach, allowing for a more precise and flexible classification of the generated solutions. Alternatively, an adaptive mechanism could be implemented, where, upon classifying a solution as infeasible, a directed mutation process is triggered, redirecting the search toward more promising regions of the solution space. Additionally, future research should explore multi-objective optimization approaches that balance cost, reliability, and computational efficiency. Expanding this methodology to real-time applications and integrating it with adaptive control strategies could further enhance its practical applicability in large-scale water distribution systems. These improvements not only open new research opportunities but also expand its potential applications in civil engineering and the efficient management of water resources.

Finally, although this study focused on a feasibility-guided integration of machine learning within the evolutionary algorithm, future work could examine alternative strategies—such as the use of predictive models to select the most promising individuals in each generation. Comparing these different integration approaches could provide valuable insights into their relative performance and suitability for large-scale water distribution network optimization problems.

## Data Availability

The datasets used and/or analysed during the current study available from the corresponding author on reasonable request.

## Declarations

### Code availability

The datasets, algorithms, and machine learning models used in this study are available from the corresponding author upon reasonable request.
